# Review—Current Concepts in Inflammatory Skin Diseases Evolved by Transcriptome Analysis: In-Depth Analysis of Atopic Dermatitis and Psoriasis

**DOI:** 10.3390/ijms21030699

**Published:** 2020-01-21

**Authors:** Julius Schwingen, Mustafa Kaplan, Florian C. Kurschus

**Affiliations:** Department of Dermatology, Heidelberg University Hospital, 69120 Heidelberg, Germany; mustafa.kaplan@stud.uni-heidelberg.de

**Keywords:** inflammatory skin diseases, transcriptomics, RNA-Seq, microarray, scRNA-Seq, psoriasis, atopic dermatitis, contact dermatitis, acne, eczema

## Abstract

During the last decades, high-throughput assessment of gene expression in patient tissues using microarray technology or RNA-Seq took center stage in clinical research. Insights into the diversity and frequency of transcripts in healthy and diseased conditions provide valuable information on the cellular status in the respective tissues. Growing with the technique, the bioinformatic analysis toolkit reveals biologically relevant pathways which assist in understanding basic pathophysiological mechanisms. Conventional classification systems of inflammatory skin diseases rely on descriptive assessments by pathologists. In contrast to this, molecular profiling may uncover previously unknown disease classifying features. Thereby, treatments and prognostics of patients may be improved. Furthermore, disease models in basic research in comparison to the human disease can be directly validated. The aim of this article is not only to provide the reader with information on the opportunities of these techniques, but to outline potential pitfalls and technical limitations as well. Major published findings are briefly discussed to provide a broad overview on the current findings in transcriptomics in inflammatory skin diseases.

## 1. Introduction

Opposed to many other tissues in inflammatory diseases, such as multiple sclerosis, the inflamed skin is easily accessible and tissue biopsies performed as whole skin punch biopsies or skin tape stripping (STS) can easily be taken. In addition, unaffected skin tissue of the same donor can be obtained as a potential non-inflammatory internal control. This enables the analysis of inflammatory skin diseases for the investigation of larger patient cohorts by means of transcriptomics. As a barrier organ, the skin builds up the first line of defense against viruses, bacteria, and fungi, and is simultaneously colonized by commensals. Therefore, the cutaneous compartment is occupied by many immune cells under steady state conditions. These cells fight infections and induce defense pathways in local tissue cells. Keratinocytes are highly active in this process. Upregulated genes support the cutaneous defense, but they also sustain pathologic conditions. In inflammatory skin diseases diverse pathogenic mechanisms take place involving immune cells and structural tissue cells. Therefore, the transcriptome of a skin lesion always reflects both the gene transcripts of the different types of infiltrated or activated immune cells as well as those of the activated local cells in the skin. As we point out beneath, this leads to a unique expression signature for specific diseases but also establishes a common cross-disease gene set among inflammatory skin diseases.

## 2. Emerging Technologies

**Figure A1:**
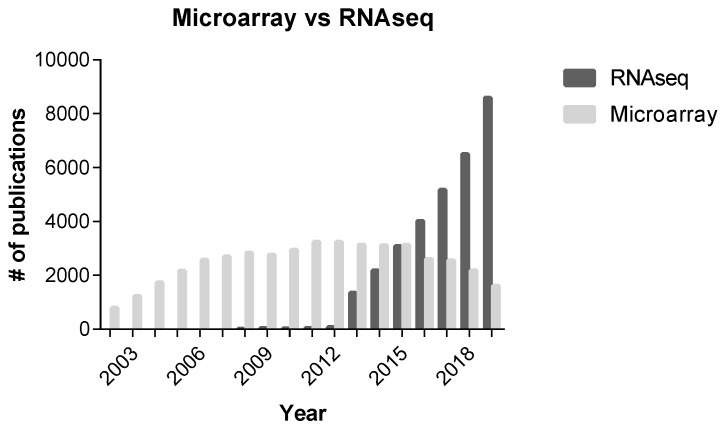
Results of a PubMed search on publication frequency of studies utilizing either microarray or RNA-Seq technology.

Microarray technology has long been used to reveal the key differentially expressed genes (DEGs) between non-involved and involved psoriatic skin [1]. The methodology was soon adapted to other inflammatory skin diseases (e.g., atopic dermatitis (AD) [2]) and inter-disease comparison [3]. In these studies, high throughput transcriptional datasets were acquired using microarray. Since 2012, studies have increasingly been focusing on techniques utilizing RNA-Seq, which is based on Next Generation Sequencing (Figure A1). Compared to microarrays, RNA-Seq shows a higher sensitivity and increased quantitative accuracy and therefore detects even low abundance transcripts. Additionally, the toolkit expands in terms of microRNA detection and alternative splicing/RNA isoform analyses [4,5,6]. Therefore, an interesting field of study evolved with emerging technologies, allowing for the classification of diseases according to their clinical phenotype and their molecular profile.

**Figure 1:**
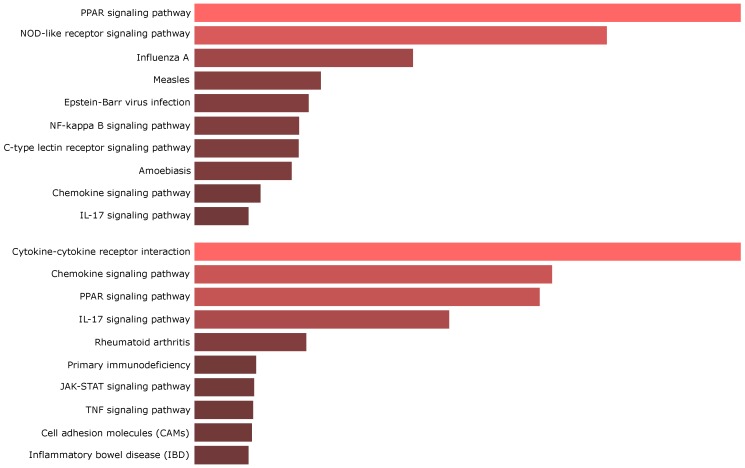
EnrichR analysis of Meta-analysis derived transcriptomes of psoriasis (MAD-5) [25] and atopic dermatitis (MADAD) [26].

With increasing simplicity and availability, comparative studies were carried out which dissect the molecular differences or commonalities of different inflammatory skin diseases. Atopic dermatitis (AD) and psoriasis (Pso) are major inflammatory skin diseases affecting 2–20% [7] and 2–3% [8,9,10] of the world population, respectively. The dissection of the molecular profiles may lead to a better understanding of the pathophysiology and involved pathways [11]. Current results of comparative studies between psoriasis and atopic dermatitis are somewhat contradictory. In some studies, a remarkable difference was seen in the transcriptomic profiles of AD and Pso, with AD rather showing similarities with other clinically characterized forms of eczema [12]. Other studies, however, rather state a largely overlapping profile using Gene Set Enrichment Analyses (GSEA) [13]. The accumulation of large datasets led to the problem of missing experimental and analytical consensus when researchers conducted meta-analytical trials [14,15]. The mere comparison of differentially expressed genes turned out to perform poorly in terms of methodology, as a high data variance with sometimes marginal overlap of genes was observed [14,16]. Comparative analyses are biased by GC contents in transcripts, abundance of expression, and transcript length [17,18]. Bioinformatic methods evolved hand in hand with technology. By utilizing GSEA, a meta-study on enriched pathways arrived at the conclusion that there is indeed consensus between different studies which is not always reflected by the mere comparison of DEGs. For example, the upregulation of important pathways such as IFNα or IL-17 signaling in psoriasis was thereby shown in transcriptomic datasets [14]. Another pitfall is reflected in several studies which reanalyzed published datasets by using the same analytical approach for each dataset. Analyzing studies by means of setting false discovery rate, fold change, and *p*-value thresholds for every study equally may be inappropriate as it neglects the respective study size and methodological differences in data acquisition [15]. With growing sample sizes, the number of detected DEGs likewise increased. Ingenuity Pathway Analysis (IPA) or similar approaches revealed the frequent upregulation of “Cancer pathways”, “Metabolic diseases”, and “Cardiovascular diseases” reflecting the characteristics of psoriasis [19,20]. For example, the upregulation of “Cancer pathways” is an interesting finding as several phenotypic features like hyperproliferative keratinocytes with a low degree of differentiation argue in this direction. One study showed the expression of *SPATS2L* in the psoriatic transcriptome which is also expressed in a leukemia cell line and is downregulated upon treatment with the Bcr-Abl tyrosine-kinase inhibitor imatinib [21]. “Metabolic diseases” fits psoriasis especially in terms of the coexistence of metabolic syndrome in psoriatic patients [22] and the presence of dysregulated lipid regulatory pathways, which are common among the top differentially regulated genes/pathways [23,24] (Figure 1).

Network construction is an intuitive way of data presentation and reflects a common approach in handling big data [27,28]. Concerning the role of associated metabolic diseases, Manczinger and Kèmeny used such a network based on a protein–protein interaction databank (STING) and chemical interaction databank (STITCH). Although further experimental validation is needed, they revealed a role of *PI3KR1* in the psoriasis interaction network [29]. This protein was previously shown to play a role in insulin resistance [30] adding further evidence to the observed differential metabolic disease pathways in psoriasis.

## 3. Ups and Downs of Skin Molecular Profiling

A major issue in many studies involves the preparation of study specimens. The choice of an appropriate body site where the skin is taken from needs to be chosen with caution due to potential differences in the skin architecture (e.g., thickness and cornification) or the inflammatory status. Additionally, as the clinical picture shows a peripheral expansion of a growing psoriatic plaque, the distance to a lesion may be of importance to capture different developmental stages of a plaque [31] or to obtain true non-lesional skin. As discussed beneath in this review, even clinically healthy non-lesional skin may not be an ideal intra-individual control due to an altered molecular state (e.g., “molecular scar” [25,32]). The use of whole-tissue biopsies guarantees preservation of the tissue complexity not just on a cellular but also on a molecular level. However, correlating the gene expression and specific cell types or tissue niches can only be modeled computationally with the help of in vitro data. Garza et al., recently presented a computational approach to deconvolute the cellular constitution of whole skin biopsies [33]. Nevertheless, this approach goes along with bias and thus potential inaccuracy. Other researchers dealt with the lack of cellular resolution by comparing transcriptomes of in vitro stimulated cell cultures with whole tissue transcriptomes. Thereby, DEGs were assigned to specific cell types [34] and investigators uncovered stimuli-related profiles (e.g., DEGs of an IL-1 profile [35]). This problem was solved by two groups which utilized laser capture microdissection (LCM) and subsequent gene expression profiling of cutaneous substructures revealing site specific profiles [36,37]. Additionally, LCM further improves the detection of low abundance transcripts by counteracting dilution effects [36].

When interpreting transcriptomic data, the structural and cellular tissue changes are important to take into consideration. Due to epidermal hyperproliferation in many inflammatory skin lesions, the dermal compartment in whole skin biopsies is underrepresented in terms of transcription products compared to healthy skin. This is supported by a study, which assigns a large set of downregulated DEGs to the dermal compartment [38]. Acquiring whole skin biopsies additionally introduces a selection bias skewing the patient collective toward older patients with a rather high disease burden [39]. A side-by-side comparison of skin biopsies and specimens taken by noninvasive skin tape stripping (STS) was performed by Kim et al. They found a significant correlation between these two methods in detecting dysregulated epidermal differentiation gene profiles, paving the way for future studies on especially younger patients and those with a milder disease manifestation [39]. Adding more value to STS specimens, hierarchical clustering identified AD non-lesional and healthy skin separately stating their differential information content [40]. The deepest penetrated epidermal layer in STS was shown to be the granular stratum which is commonly lost in psoriatic lesions [39,41]. Accordingly, due to structural differences of the skin in both diseases, distinct molecular profiles are expected when comparing psoriasis and AD skin.

Nevertheless, by preserving the complex molecular structure, the clustering of detected differentially expressed genes enables pathway analysis as well as network construction (e.g., weighted gene co-expression network analysis (WGCNA)). These tools identify upstream acting master regulatory factors (e.g., transcription factors) and visualize biological meaningful interactions intuitively [28,42,43,44]. Assessing the network of expressed transcription factors may provide information about the cellular infiltrate and the respective polarization (e.g., Th1–T-bet) [24,45]. Network construction can also be used to expose protein–protein interactions or to predict a potential drug effect based on publicly available datasets as described by Manczinger and Kèmeny [29] and others [44,46]. Ultimately, to gain biologically relevant insights via high-throughput methods, considering genomic information in the course of the data analysis is indispensable and reveals relevant DNA motifs/“psoriasis responsive elements”. This approach may also lead to the detection of new psoriasis susceptibility loci [47].

Generally, “omic” technologies heavily rely on data science to handle and extract interesting patterns from large sets of measurement points. A recent study introduced the concept of a multi-omic clinical workflow to identify treatment responses in a pilot cohort of psoriasis patients treated with the TNF inhibitor etanercept [48]. Another multi-omic approach included a highly diverse patient collective to investigate low-frequency and rare-coding variants associated with AD to improve the understanding of disease heredity. Through this in-depth view, they found a new and potentially therapeutic target comprising the surface receptor CD200R1 and its associated adaptor protein DOK2 [49].

## 4. Transcriptomic Insights into Disease Mechanisms

### 4.1. Disease-Related Findings

#### 4.1.1. Psoriasis

During the last decades, different features of the psoriasis pathogenesis have been illuminated by focusing on either an innate immune system centralized viewpoint or a more prominent role of T helper cells focusing on Th1 polarization [50,51]. The latter perspective evolved from xenotransplant models in which stimulated lymphocytes were needed to induce a psoriasis-like phenotype arguing against the role of the innate immune system alone [52]. In line with this, an aberrant T cell functionality leads to an atypical cytokine milieu within the affected skin site. Whether psoriasis results from dysfunctional immune cell inherent properties is still under debate. Case studies, which argue in favor of this hypothesis, reported the dissolvement of psoriatic plaques after allogenic stem cell transplantation. This is further supported by studies on the role of hematopoietic stem cells in psoriasis [53]. Keratinocytes were assigned a non-passive role in this concept by responding with hyperproliferation, impaired differentiation, and feedforward acting proinflammatory cytokine release. The evaluation of DEGs revealed that the molecular pathways found in psoriasis correspond to the observed histological features. A prominent role was shown for the highly differentially expressed “STAT1-57” pathway, which includes proinflammatory signaling paired with NFκB activation and inhibition of apoptosis [54]. Genes correlating with the rather immature state of the keratinocytes in psoriasis, like *ESR1*, encoding for the estrogen receptor α which inhibits apoptosis, were found to be differentially expressed [55]. A comparative study between psoriatic transcriptomes and defined expression data from in vitro stimulated keratinocytes showed a large gene expression overlap between IL-1α stimulated keratinocytes (innate immune response) and psoriasis transcriptomes. However, such a connection could not be found for IFNγ-stimulated keratinocytes (mixed adaptive and innate immune response), which suggests a prominent role of the innate immune system [56].

In line with a keratinocyte-centralized view of the pathogenesis of psoriasis [57], keratinocyte-specific DEGs (e.g., *SERPINB4*, *S100s*, *TCN1*, *KRT16*) [58] and *IL1* family cytokines (e.g., *IL1F5*/*6*/*8*/*9*) [59] are highly prevalent in lesional skin. Therefore, a major aim of psoriasis treatment may be to intervene in the innate milieu induced by “stressed keratinocytes”, which in turn is potentially triggered by danger signals (e.g., ATP [60]). However, one should bear in mind that the comparison of single-cytokine stimulated cell cultures may be difficult to compare with whole tissue transcriptomics. In addition to obvious reasons regarding the gradient of complexity from in vivo to in vitro systems, cytokine responses often show a high redundancy. Although the accordance may not be as prominent in terms of overlapping DEGs, similar pathways may be activated in vivo and in vitro [61].

As the role of IL-17 and IL-22 in psoriasis became apparent, hypotheses about dual secreting T cell phenotypes emerged [62]. Publicly available datasets were analyzed by unsupervised clustering using t-Distributed Stochastic Neighbor Embedding (t-SNE) dimensionality reduction. Although the analyses of Le et al. support the idea of a close relationship between IL-17A and IL-23, their results are rather indicative for separate IL-17A- and IL-22-secreting T cells instead of a dual secreting phenotype [63].

A potential role of neutrophil extracellular traps (NETs) in IL-17 release by neutrophils and mast cells was proposed, as these cells are highly prevalent in psoriatic lesions [64]. Lambert et al., investigated the effect of NETs on PBMCs stimulated via CD3/CD28. Despite the artificial character of the employed system, their findings indicate that NETs contribute to the Th17 polarization as assessed by the DEGs induced by NET exposure [65]. Research on the etiology of psoriasis includes the investigation of potential autoantigens (see, e.g., in [66]) and specific phenotypes of professional antigen presenting cells (APC). Inflammatory dendritic cells, including CD11c+CD1c- resident dendritic cells, were suggested to be key players in the pathogenesis of psoriasis. Through the production of mediators classically found in psoriasis, these cells reflect pathognomonic cell populations [67]. In contrast to CD1c+ DCs, the CD11c+CD1c- DC population, which is specifically present in chronic skin inflammation, was shown to express S100 proteins, TRAIL, iNOS, and both IL-23 subunits (IL-12p40 and IL-23p19) [68]. These cells were made responsible for the increased iNOS levels specifically seen in psoriatic lesions and were stated TNF-/iNOS-producing DCs (Tip-DCs) [69]. Alternatively, CD163+ macrophages were also shown to harbor this effector function. However, the in vivo distinction of a rather DC-like state versus a macrophage-like state is nontrivial, and the role of Tip-DCs/macrophages is under debate [70,71]. Although a single prominent cell type might be responsible for a certain cytokine response in a specific disease state, the cytokine milieu is sustained by various contributors (e.g., Langerhans cells, inflammatory dendritic cells, and mast cells [72,73,74]).

A potential contribution to an aberrant cell status was shown for several rapid TNF inducible proteins TNFAIP3/6/8, which are produced by inflammatory DCs. Tolerance promoting and therefore chronic inflammation inhibiting functions were shown for TNFAIP3/8, whereas TNFAIP6 was reported to act proinflammatory. In line with this, TNFAIP3/8 were found to be downregulated on transcriptional level in lesional psoriatic skin [75]. In addition to classical antigen presenting cells, currently neglected cell types such as myoblasts may be important in disease pathomechanisms as well [76].

In the pre-biologic era, IFNγ was tested for the treatment of psoriasis. The subcutaneous IFNγ injection led to the development of psoriasis-like lesions, which suggested a potential role of IFNγ in the pathophysiology of the disease [77]. Furthermore, in the course of systematic investigations common molecular feature were detected (e.g., through unsupervised clustering) [78]. Interestingly, keratinocytes in lesions showed a high expression level of antiviral response genes, which potentially increases the IFNγ secretion by incoming immune cells [54].

An antiviral response in psoriasis was investigated by Raposo et al., who provided evidence for reduced RNA editing activity in lesional skin. This decrease in adenosine-to-inosine editing entails an accumulation of unwinded dsRNA in keratinocytes with downstream activation of the MDA5/MAVs pathway [79]. In line with this, dsRNA pattern recognition sensors were shown to be upregulated in psoriasis [80]. In addition, the upregulation of *CH25H* expression, transcribing for an enzyme, which contributes to an oxysterol biosynthesis pathway by producing 25-hydroxycholesterol from cholesterol, reflects the increased potency of an antiviral response, as increased CH25H levels are associated with a reduced viral entry [81,82]. Although not frequently reported, this upregulation may also be present in lesional AD [83] and allergic contact dermatitis (ACD) [84]. Generally, the downregulation of many other lipid biosynthesis enzymes promotes the unique role of CH25H [85]. In terms of substrate feedforward activation, the increase in 25-hydroxycholesterol may also account for the enhanced *CYP7B1* expression as revealed in a comparative study on CYP450s in psoriasis and melanoma [86]. Interestingly, the TLR7/8 agonist imiquimod (IMQ) also signals via IRF3/7 and thereby induces an antiviral response in the respective psoriasis disease model [87]. Strikingly, the opposite was found in atopic dermatitis patients with eczema herpeticum where *IRF*s were downregulated in PBMCs [88].

The proinflammatory environment in psoriatic lesions induces peroxisome proliferator-activated receptor (PPAR) transcriptional regulators. This activation is accompanied by altered intracellular lipid molecules which in turn feedforward the PPAR pathway [89]. Strikingly, the expression of proinflammatory *CCL20*, *IL8*, and *S100A7* was strongly reduced by cholesterol depletion in IL-17A stimulated keratinocytes [85]. The expression of *PPAR*s may therefore also be relevant in terms of the therapeutic usage of glitazones or inhibitors of lipid synthesis pathways [29]. In line with accumulating intracellular lipid molecules, members of the sterol regulatory element-binding protein (SREBP) family were downregulated in IL-17A stimulated keratinocytes and lesional psoriatic skin [85]. The SREBP downregulation was also observed in skin of AD patients, which implies the existence of a common feature in psoriasis and atopic dermatitis [90].

#### 4.1.2. Atopic Dermatitis

Transcriptomic studies on atopic dermatitis have been focusing on the concept of a dysregulated, e.g., Th2-like, immune response [91], and the role of cutaneous barrier abnormalities [92]. In this respect, the dysregulated key epidermal genes filaggrin (*FLG*) and loricrin (*LOR*) were grouped as members of the epidermal differentiation complex (EDC) [90,93,94], which is encoded on the 1q21 susceptibility locus [95]. Factors of the EDC are often antimicrobial effector molecules of the innate immune response [96]. A decreased expression of these proteins was found in AD compared to psoriasis [97]. This may be functionally linked to the specific constitution of the immune cell infiltrate [98]. Mutations of the epidermal structural protein FLG are a main finding in AD supporting the outside-in hypothesis, which focuses on the barrier dysfunction and environmental factors as main disease drivers. Unsupervised clustering revealed the similarities in transcriptomic characteristics of extrinsic AD and patients with FLG mutations [93]. However, FLG mutations are not commonly seen in AD, questioning their impact on disease induction [99]. Furthermore, the comparison with ichthyosis vulgaris (IV), which is regularly accompanied by FLG mutations, seems to argue against the outside-in hypothesis as no excessive inflammation is seen in these patients [100].

The maintenance of the skin-barrier function relies heavily on homeostatic conditions sustaining the equilibrium between cellular motility and excessive tissue destruction. Proteases are commonly under the top dysregulated genes in AD transcriptomic profiles [15,83]. Loss of the serine protease inhibitor Serpin3a in mice was shown to induce the upregulation of proinflammatory pathways after a single patch of Aspergillus fumigatus extract on the skin of transgenic mice. On the other hand, the protease inhibitor-deficient mice also showed decreased trans-epidermal water loss (TEWL), which is commonly increased in AD skin [101]. Tissue homeostasis in inflammatory skin diseases therefore relies on a fine-tuned interplay of proteases and anti-proteolytic acting factors (e.g., PI3/elafin) to maintain skin-barrier function [102,103]. Besides epidermal disturbances in structural protein expression (FLG), excessive proteolytic activity (e.g., regulated through SERPINs), dysregulated lipid content and composition, as well as altered aquaporin expression with a broader distribution in several epidermal layers may also contribute to the fluid loss [104].

Research on the transcriptomic profile in AD is mainly conducted using whole skin biopsies. However, Esaki et al., concisely showed compartment specific transcriptional profiling using laser capture microscopy [36] as many other studies are biased in terms of architectural changes in skin compartments in inflammation compared to healthy skin [93]. For example, although *IL34* and EDC members (e.g., *S100A7*/*8*/*9*, *PI3*, *SPRR1A*, and *CLDN*s) were specifically expressed by the epidermis, dermal transcriptomics revealed the expression of lymphoid tissue homeostatic systems (e.g., *CCL19*, *CCL21*, and *CCR7*) [36], which are relevant in immune cell cluster formation, termed as skin associated lymphoid tissue (SALT) [37,84,105].

The aberrant differentiation profile also leads to ectopic olfactory receptor expression. Increased mRNA levels of *OR10G7* were recently detected by skin tape stripping in atopic dermatitis patients in the upper epidermal layers. Although this may constitute a potential mechanism of odorant-induced skin inflammation in AD patients [106,107], an earlier study found olfactory receptor genes to be downregulated in AD [40]. This may reflect the above-mentioned difficulties in inter-study comparison or may argue for the high diversity seen in the AD pathophysiology. In addition to localized skin manifestations, cutaneous inflammatory diseases are commonly accompanied by systemic pathological features [26,108,109]. The complex interplay was also revealed by studies investigating the connection between food allergy or oral tolerance and atopic dermatitis [110,111], as discussed beneath in this review. In order to acquire the full picture of the association between localized skin and systemic features, a side-by-side study of different body compartments would be necessary. So far, most studies rather focused only on PBMCs [2,112,113,114] or on skin-derived specimens.

Commonly reported dysregulation of the lipid metabolism pathway is not just a feature in psoriasis [115], but also frequently seen in AD and negatively correlates with the “immune response” pathway in the Gene Ontology (GO) enrichment analysis [15,26,90]. Major genes involved in this pathway are present in the polyunsaturated fatty acid pathway (PUFA) and are responsible for proper cellular membrane constitution (e.g., *FADS1*/*2*, *ELOV5*) [90]. The disturbed lipid profile contributes to barrier disturbance and may also influence cellular signaling directly by impaired lipid raft constitution [116].

As mentioned above, diverse specimen acquisition chosen of a heterogenous study cohort reflecting the full clinical picture is necessary to prevent biases. This is especially observed in AD studies. A comparative investigation showed a rather low Th17/IL-23 contribution in adult AD patients whereas Th1 profiles were observed in sustained lesions [117]. This does not hold true for pediatric patients. Brunner et al., found a lack in Th1-, but enriched Th17-/Th22-associated gene profiles (e.g., *CCL20*, *IL23*, *IL36*, and *IL36RN*) in pediatric patients. This observation was additionally accompanied by psoriasis-like expression of antimicrobial peptides. Moreover, TEWL pointed to a skin-barrier disruption which was independent of altered expression of *LOR*, *FLG*, or *LCE*s [118]. Another bias was detected by comparing AD manifestation among different ethnicities. While European American patients rather suffered from a Th1-skewed AD profile, the Th17 axis was increased in African American and Asian AD patients, similar to the findings in pediatric patients [119].

#### 4.1.3. Other Inflammatory Skin Diseases

Although most studies have been focusing on psoriasis and AD, other inflammatory skin diseases were also analyzed for transcriptional changes. Acne vulgaris is considered as a disease of the pilosebaceous unit with strong contributions of *Propionibacterium* (*P.*) *acnes* [120]. The analysis of the microbial microarray data reveals the presence of different strains and is useful in studying host interactions by the analysis of differentially regulated pathways. Through analyzing bacterial metabolic pathways, this approach led to a potential mechanism of supplemented VitB12-induced acne [121]. WGCNA was utilized to construct interaction networks which highlight the role of central cytokines in the disease’s pathophysiology (e.g., *IL1β* and *CXCl1*/*2*) [122,123]. Recently, the role of miRNAs was also introduced into this research field as depicted in Table A2 [124,125].

### 4.2. The Itch-Related Transcriptome

Pruritus is an important symptom in inflammatory skin diseases contributing significantly to the impaired quality of live in affected patients. Transcriptomic analysis revealed an itch-associated molecular profile which was appropriately termed “itchscriptome” [24]. Common DEGs included *IL31* [126,127], TRP channels (e.g., *TRPA1* and *TRPV2*) and neuropeptides [128]. This already states the high relevance of histamine-independent itch in AD. It is mediated by a functional link between immune cells and free nerve endings mainly via IL-31, which in turn induces neuropeptides like BNP [126,128]. The connection of the nervous system and chronic inflammatory skin disease brings up the question to what extent neurogenic inflammation accounts for disease manifestation and characteristics (e.g., chronicity, physical and psychological stress-induced manifestation) [126,129]. Interestingly, whereas TRP channel upregulation was also seen in psoriatic skin, the receptor type was disease specific, respectively (i.e., *TRPV3* and *TRPM8* in psoriasis only) [128].

Non-histaminergic itch is additionally important in allergic contact dermatitis potentially mediated via thymic stromal lymphopoietin (*TSLP*) [130]. Given the allergen-dependent immune response characteristics which are discussed in this review, allergen-specific murine models are needed in translational research to reach high validity.

### 4.3. Disease Subgrouping

Disease variations are often classified according to their respective clinical phenotype including symptoms and physician-determined signs. To reach a high inter-patient consistency in valid disease classification, studies integrated molecular findings to increase accuracy and render the categorization process examiner-independent. Clustering analysis revealed the molecular heterogeneity of psoriasis as different patients did not cluster together and rather showed a dissimilar distribution [131]. This approach in combination with clinical data may be promising in detecting different disease phenotypes and in customizing therapies. By considering the degree of fold change in genes between different patient samples, investigators are enabled to classify disease types according to the severity level. A “low-level” or “high-level” inflammatory status is seen in patients showing a weak or strong IFNγ profile. This reflects the heterogeneity of molecular psoriasis classification [58]. Correlating the clinical PASI score with transcriptomic data separated “mild” and “severe” psoriatic transcriptomes and revealed the existence of increased immunoregulatory mechanisms. Moreover, a more pronounced Th1/Th17 profile in mild psoriasis was detected [132]. The lack of immunoregulatory activity leading to chronic plaque manifestation is a tempting hypothesis [133].

Connecting fold change and clinical phenotype was also shown in the example of thick and thin plaque psoriasis, which overlap to a large extend in their transcriptomic profiles [82]. Similar results were found in comparing skin and scalp psoriasis [134]. The phenotypically severe psoriasis form generalized psoriasis pustulosa (GPP) exhibited a strong granulocyte chemotactic transcriptome reflecting the disease’s histological findings (e.g., subcorneal neutrophilic pustules and neutrophilic spongiosis) [135]. Marrakchi et al., presented the role of *IL36RN* mutation in a case of hereditary GPP and suggested a strong role of IL-1β and IL-36 in the disease pathophysiology [136]. Additionally, the IL-1R antagonist anakinra shows frequently incomplete treatment response and IL-36 may be a more promising druggable target. However, despite GPP-specific features and susceptibility factors (e.g., *CARD14* and *AP1S3*) [135], *IL17* mRNA is also constantly present in pustular lesions. In line with this, unsupervised clustering revealed an overlap between plaque psoriasis and GPP. Taken together, generalized pustular psoriasis exhibits a skewed profile toward innate immune responses with simultaneous IL-17 expression. These findings justify the clinical subgrouping of GPP as a psoriatic skin disease, as GPP rather displays a molecular facet of psoriasis than a completely different disease [137].

A more recent study compared conventional, scalp and palmoplantar psoriatic transcriptomes and described a core set of regulated genes common to all of them [138]. Especially relevant in scalp psoriasis, a single study investigated the observed hair loss seen in this clinical type. By creating a sebaceous gland gene signature, they argued for a sebaceous gland atrophy supported by findings in transcriptomes of *Scd1* knockout mice and psoriatic lesions. This profile may be useful in future general hair follicle studies [139] and acne research as this disease is focused on the pilosebaceous unit [121]. The clinical distinction of common disease phenotypes is additionally supported by reviewing the main differentially regulated pathways [138].

The discrimination of pustular palmo-plantar psoriasis (PPPP) and palmo plantar pustulosis (PPP) is under ongoing discussion. Comparative transcriptomics of pustular diseases such as GPP, PPP and acute generalized exanthematous pustulosis (AGEP) revealed their converging effector pathway which leads to neutrophilic inflammation [140]. Objective classification guidelines are missing and physician-dependent. By assessing the transcriptomics in comparison to healthy acral skin and in contrast to plaque psoriasis (PP), PPP, and PPPP clustered together in unsupervised dimensionality reduction (e.g., t-SNE, PC) but accumulated distinct to healthy skin or PP [141]. PPP and PPPP may therefore describe a spectrum of a common disease entitiy.

Even more intriguing may be the approach of transcriptome-guided disease classification. In atopic dermatitis, different molecular mechanisms, so called endotypes, are known to be responsible for the disease manifestation according to the observed patient cohort. In line with the outside-in hypothesis of external environmental allergens as the main disease driving cause inducing an IgE response, Martel et al. and others classified patients into IgE high extrinsic AD (ADex) and IgE low intrinsic AD (ADin) (i.e., outside-in and inside-out hypotheses) [142]. Interestingly, they observed a clustering of transcriptomes of ADin with mild psoriasis specimens and a separate cluster of ADex supporting the idea of different endotypes [93], which suggests the existence of a heterogenous disease group. Current clinical practice classifies AD as a single disease. The concept of AD endotypes and their therapeutical implications have also been nicely reviewed by Czarnowicki et al. [143].

A severe complication in atopic dermatitis patients is the occurrence of HSV-1 infection which clinically manifestates as eczema herpeticum (“ADEH+”). The investigation of HSV-1 stimulated PBMC response of either ADEH+ or ADEH- patients resulted in distinct populations in unsupervised clustering of the respective transcriptomes. Type I and III interferons were shown to be downregulated in the ADEH+ group. This downregulation may be rather due to changes within the upstream regulatory layer, as the master regulatory factors *IRF3*, *-7*, and *-9* were downregulated as well [88]. This was supported in an eczema herpeticum mouse model established by Kawakami et al. They reasoned in favor of a decreased natural killer (NK) cell function due to impaired type III interferon expression which in turn promotes HSV-1 spreading in eczematous conditions [144]. Other factors in HSV-1 susceptibility of some AD patients were shown to include *FLG* and ankyrin repeat domain 1 (*ANKRD1*) mutation status. FLG expression negatively correlated with an antiviral response and haploinsufficiency increased the risk of eczema herpeticum [145]. In contrast, *ANKRD1* was found to be downregulated in ADEH- patients, suggesting a reduced antiviral response via diminished *IRF3* signaling [146].

By investigating associated comorbidities this potential grouping criterion was also seen in AD and food allergy. Recently published, Leung et al. investigated AD children with or without food allergy (FA+/FA-) compared to non-atopic (NA) children. By analyzing the transcriptome of STS specimens, they revealed the distinct dysregulated *KRT* expression in FA+ versus FA- or NA (i.e., *KRT5*, *-14*, and *-16*). Furthermore, they uncovered a decreased content in ceramides and a pronounced Th2 cytokine profile. The excessive TEWL suggests a higher degree of barrier disturbance in FA+ patients, which is potentially responsible for an increased allergen penetration and disease exacerbation. It is therefore crucial to improve the barrier function in a primary and secondary disease setting through topical agents. This treatment approach might be especially beneficial in FA+ patients [110].

As skin is a major body site directed toward environmental impacts, the microbiome is a crucial player in the tissue homeostasis and fine-tuned regulation between immune activation and tolerance is necessary [147]. A recent study revealed the existence of different endotypes according to the microbiome and revealed a high diversity of microbial species in psoriatic skin [148,149]. As dysregulated EDC members often show antimicrobial activity (e.g., *S100*s and *LEC*s), the investigation of the microbiome in dependency of genetic mutations would be intriguing [150]. In contrast, the skin microbiome of AD patients was highly over-represented by *Staphylococcus* (*S.*) *aureus* which allowed the discrimination of a *S. aureus* “low” and “high” colonized state. The latter one was shown to be paralleled by increased barrier dysfunction (e.g., *CLDN8*, *FLG*) and a stronger immune cell related profile (e.g., *IL13*, *IL5*) [148,151]. This identified “high” colonized state may also be more prone to HSV-1 infections as it is seen in ADEH+ patients [88]. Surprisingly, Kennedy et al., conducted a prospective study on the developing microbiome and its impact on AD onset in children. They found that *S. aureus* was literally absent in infants which developed AD within the first year of life [152]. However, additional studies are needed to clarify the direction of this tempting idea of a causal relation. The environmental context, including the microbiome, may drive skin inflammation toward a specific characteristic by influencing pathophysiology (e.g., impact of *S. aureus* toxins [127,153,154]), as well as treatment response and prognosis [155]. Another example of AD diversity supporting the concept of multiple endotypes was stated in a study by Dyjack et al., who found non-lesional skin with either strong (“AD high”) or weak (“AD low”) immune cell infiltration. They showed the correlation of an “AD high” -type with clinical severity by clustering of “AD high” with lesional skin samples [40]. The identification of these endotypes (e.g., *S. aureus* low/high, AD low/high) may allow for patient-stratified therapy and prognosis. The clinical decision making has therefore to rely on data derived from molecular profiles and genetic association studies, which were correlated to patient-relevant features. Through conceptualization of this approach and integration into clinical classification systems, adequate single patient-adapted treatment is conceivable.

Contact dermatitis is currently divided according to the pathophysiology into irritant (ICD) and allergic contact dermatitis (ACD) [156]. A different approach may be a molecular profile-adapted classification showing improved criteria for treatment and prognostics decision making. To date, in particular, ICD studies [157] and correlative studies including both contact dermatitis entities are needed. Dhingra et al. investigated the induced immune reaction in response to different common allergens in a human study. They presented an allergen-dependent T effector cell polarization with a Th1/Th17 response to nickel, Th2 response to fragrance and a Th17/Th22 response to rubber. This differential pathomechanism emphasizes the heterogeneity of ACD and contrasts to the principle of a discrete disease. Recently, a Th2/Th17 profile was shown for a poison ivy ACD response in mice compared to a oxazolone-induced Th1-prominent polarization. Poison ivy ACD is in contrast to many other established antigens highly relevant in the human disease [130]. Therefore, the classical view of Th1/Th2-skewed profiles shifted toward a more prominent role of the Th17 axis. Molecular profile-based classification therefore strongly argues in favor of an allergen-dependent ACD disease grouping [84] and allergen-stratified therapeutic approaches may show superior patient outcomes.

### 4.4. Emerging Possibilities in Disease Studies

Transcriptomic evaluation allows for identification of all kinds of RNA molecules [4,5,18]. Several studies focused on the role of micro RNAs (miRNA) in inflammatory skin diseases (Table A2) [18,158,159,160]. MiRNAs are short oligonucleotides that regulate the cellular status on the transcriptomic level [161]. As miRNA profiles clustered diseased and healthy patients separately [162], their potential competence in reflecting the course of a disease renders them as putative biomarkers. In this regard, additional investigations of miRNAs in response to treatment are needed [163].

The analysis of the differential exon usage reflects another interesting analytical approach [6]. The in-depth view through RNA-Seq enables alternative splicing analyses which revealed the existence of specific RNA isoforms present in non-lesional and lesional psoriatic skin (e.g., *ETV3_3*, *NLK_6*, long isoform of *S100A7A*) [5]. A set of involved splicing events seems to be conserved among mice and humans highlighting its biological significance [164]. Utilizing weighted gene co-expression network analysis, long non-coding RNA (lncRNA) molecules were shown to be highly abundant in the psoriatic [165] and AD transcriptomes [166]. This high abundance paired with the observed treatment response potentially provides a molecular surrogate marker for successful treatment [107].

In another interesting study, the high sensitivity of RNA-Seq datasets was exploited to gain a closer look into the T cell receptor pool in diseased specimens. Distinct receptor segments were correlated with skin status: *TRAJ39* was strongly underrepresented in psoriatic skin compared to healthy skin, whereas *TRAJ23* was upregulated. Other differentially expressed segments included *TRBV3-1*, *TRBV3-2*, and *TRGV5*. Importantly, this regulation was specific to psoriasis as compared to atopic dermatitis [167]. This analysis could harbor high potential in psoriatic antigen research given its predictive potential by correlating known antigens with T cell repertoires [168,169]. Additional integration of microbial data is going to show interesting associations as well and may further conceptualize the link between T cell effector phenotypes and disease characteristics [170].

### 4.5. Molecular Profiling Reveals a Pre-Inflammatory State

**Figure 2:**
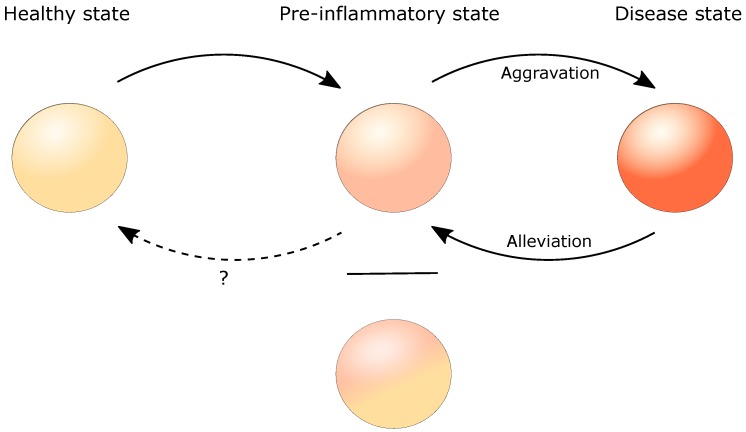
Hypothesis of an pre-inflammatory state as described by the molecular profile in inflammatory skin diseases. The presence of a “molecular scar” may represent an alternative baseline of the transcriptome.

**Figure 3:**
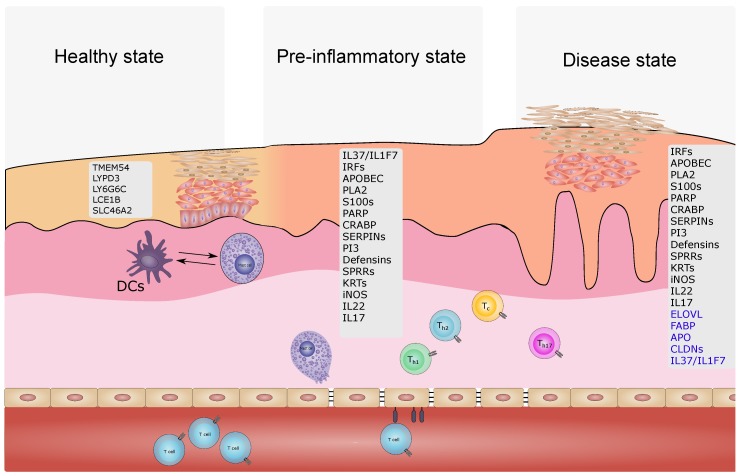
Development of psoriatic lesion on the basis of the proposed model of a multistage process including healthy—pre-inflammatory—disease state. Genes in healthy [38], non-lesional, and lesional psoriatic skin [19,25,37,81,172]. Gene symbols marked in blue represent downregulated genes.

The idea, that non-lesional skin from psoriasis patients may show structural and/or molecular differences compared to skin of not affected individuals, was supported in a xenotransplant model. A psoriatic phenotype developed from xenotransplanted non-lesional skin whereas no lesions developed in transplanted healthy skin [171]. Investigating the psoriatic transcriptome in the continuum healthy—non-lesional—lesional skin revealed an intermediate molecular profile, the pre-psoriatic state (Figure 2 and Figure 3)

While certain genes are expressed in lesional and non-lesional skin (e.g., *IL36G* and *IL36RN*), others are only expressed in either form (lesional only: e.g., *SPRR*s, *SERPIN*s, *S100*s; non-lesional only: *IL37*) [5,172]. At the same time, *IL37* was shown to be downregulated in lesional skin by others [173]. The existence of these three states seems to hold true for scalp psoriasis as well [134]. As a hallmark of psoriatic skin, abnormal vascular formation can be found in terms of general quantity but also abnormal configuration with twisting hyperpermeable blood vessels. This is supported in transcriptomics by the involvement of proangiogenetic genes which are already expressed in non-lesional psoriatic skin [174]. Dermal mesenchymal stem cells are discussed as potential contributors in regulating angiogenesis in the skin [175,176,177,178]. In addition, anti-TNFα treatment led to the upregulation of *VEGF* negative regulating pathways [179]. Notably, the markedly diminished RNA editing functionality in lesional skin was not observed in non-lesional skin [79]. Skin tissue-resident T memory cells (Trm) are thought to contribute to the underlying mechanism of a consistent pre-psoriatic skin state. In ex vivo healthy and psoriatic skin transplant analyses, differential cytokine responses of diseased transplants stimulated with OKT-3 were assigned to Trm. The disturbed tissue homeostasis is reflected by a skewed cytokine profile toward IL-17 secreting Trm [180]. A recent study identified pre-lesional changes also in purified primary keratinocytes reasoning for a differential gene expression program in this cell type [181]. Whether these changes are causal for disrupted tissue homeostasis and a keratinocyte inherent feature or rather the effect of an underlying disturbed system is unclear.

This skin state continuum holds also true for atopic dermatitis suggesting a barrier defect or dysregulated immune response, which drives the increased susceptibility of non-lesional AD skin. The skin status is thereby prone to transform into lesional skin by a variety of endogenous (e.g., distress) or exogenous trigger (e.g., dust and microbes) [182]. For example, although Malassezia sympodialis is considered as a skin commensal fungus, treating non-lesional AD skin in a patch test with Malassezia sympodialis leads to a similar gene expression profile as observed in lesional AD contrasting the effects observed in healthy skin [90].

## 5. Cross-Disease Studies Define a Core Molecular Profile

Medical classification systems of diseases increasingly include molecular data [183]. This potentially improves the treatment since responders may be distinguished from non-responders. Furthermore, subtypes of a certain disease may be resolved, although the clinical discrimination is not possible or predictions regarding the prognosis are depending on molecular features. One aim of research on transcriptomics in diseases is the evaluation of a specific set of genes reflecting the core characteristics of a disease on a molecular level. This core transcriptome may thereby help to classify a possibly clinically heterogeneous disease into a scheme which may be valuable due to the above-mentioned reasons. Meta-analytical approaches tried to come up with this core gene set of psoriasis [25] and atopic dermatitis [15,26]. To evaluate the smallest set of genes in order to classify a lesion from non-lesional psoriatic skin, Tian et al. used an earlier published algorithm by Huang et al., and defined a set of 20 genes [25]. This approach is potentially going to assist in the usage of large datasets in combination with computational modeling [184] and machine learning algorithms which are increasingly simplified so that “wet-bench scientists” and clinicians can take advantage of it [185]. For example, a recent study focused on the evaluation of a variety of datasets from transcriptomic studies including several forms of psoriasis, atopic dermatitis, lichen planus, and contact dermatitis to train a classifier using four genes only (*IL36G*, *CCL27*, *NOS2*, *C10ORF99*) [186].

The Meta-Analysis Derived Atopic Dermatitis (MADAD) transcriptome by Ewald et al., includes important genes commonly seen in AD transcriptomics. This comprises genes of the EDC (e.g., *S100A*s, *PI3*, and *CLDN8*), lipid metabolism genes (e.g., *ELOVL3* and *FABP7*), and genes encoding for proteases (e.g., *MMP*s) [26]. Gosh et al. determined another AD core transcriptome termed 89ADGES [15]. Tsoi et al., conducted a study regarding the comparison of atopic dermatitis and psoriatic transcriptomes. Their data emphasize the existence of a common skin inflammatory gene set and point to correlative aspects between non-lesional psoriatic skin and atopic dermatitis skin. On the other hand, using a small gene set consisting of *IL17A*, *IL13*, and *IL36G*, their classifier identified the diseases almost perfectly. This supports the presence of distinct features [187]. This finding is consistent with investigations by Kamsteeg and colleagues. They presented the overlap of eczematous diseases transcriptomics (e.g., AD and contact dermatitis) and segregated cluster formation from psoriasis [12]. Interestingly, the AD—psoriasis common gene set determined by Choy et al., is skewed toward a neutrophil attracting profile [188], which may be due to the underlying commonalities in IL-17 signaling.

Inter-disease studies often neglect the existence of several endotypes, especially seen in AD. As such, classification systems may be only useful for the identification or discrimination of two disease states or entities in a specialized manner. The above-mentioned identification of psoriasis and AD according to a specific gene set might work in case of a certain AD type showing strong transcriptomic differences but might be otherwise rather poor in performance. For example, intrinsic AD in contrast to extrinsic AD showed upregulation of psoriasis-typical genes (e.g., *IL22*, *IL36G*, *CCL19*, and *CCL22*) complicating discrimination of intrinsic AD and psoriasis [93]. Besides the similar molecular profile, quantitative differences in expression levels may add important information to overcome this issue as seen in the IL-36 expression which is more prominent in psoriasis [18]. Although psoriasis is a bona fide IL-17-dependent disease, allergic asthma and atopic dermatitis are commonly viewed as classical Th2 driven diseases [189]. However, transcriptomic profiling revealed the existence of different endotypes in the latter diseases. In allergic asthma, IL-17- and IL-13-driven endotypes aggregated separately in an unsupervised clustering analysis and the two profiles seemed to be exclusive. In addition, this led to the identification of smoking as a risk factor specifically for the IL-17-driven type [190].

**Figure 4:**
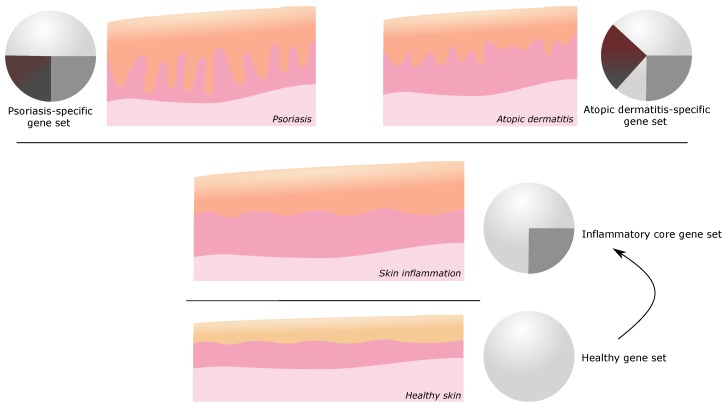
Illustration of the concept of a common/core gene set of inflammatory skin. A psoriasis- or atopic dermatitis-specific gene set defines the respective disease state and phenotype.

To dissect disease specific core transcriptomes, comparative studies are necessary. Furthermore, by combining the findings of several inflammatory skin diseases, a common transcriptional gene set of skin inflammation became apparent (Figure 4). The comparison of discoid lupus erythematosus (DLE) and psoriatic lesions revealed a differential clustering upon dimensionality reduction although a certain overlap was observed pointing toward the existence of a common cross-disease profile. Through gene set enrichement analysis, the differential T cell polarization toward Th17 in psoriasis and Th1 in DLE was supported [191].

Although most psoriasis-specific DEGs in skin were found in psoriatic keratinocytes [192], PBMCs are an easily accessible specimen which may provide additional insights. Indeed, PBMCs from psoriasis patients show a dysregulated transcriptomic state [193,194,195]. Tan et al. noted the potential role of complement factors by comparing psoriasis and rheumatoid arthritis [196]. Notably, keratinocytes were shown to express the complement C3a receptor, which may contribute to the innate response in early skin inflammation [197]. Laborious clinical diagnostics may could be simplified by validating PBMC analysis for this purpose. For example, patch test is considered the gold standard in allergic contact dermatitis (ACD) and PubMed results go back until the mid-20th century. With transcriptomics, the possibility of developing a fast testing workflow using PBMCs emerged [198].

As before-mentioned, FLG is considered to be an integral contributor of the skin-barrier. However, the inter-disease comparison with ichthyosis vulgaris, which is mostly accompanied by *FLG* mutations, challenges this hypothesis [199]. The excessive inflammation and upregulation of xenobiotic metabolizing pathways are missing in IV, which is contradictory to the outside-in hypothesis on the basis of filaggrin mutations [100]. In addition, the frequency of *FLG* mutations in a US cohort of children with AD was approximately 16%, showing that this genetic variation only accounts for a subpopulation of patients [99]. *FLG* mutations were as low as 3.2% in an African American AD population, although this group presented with increased features of an extrinsic AD profile (i.e., higher IgE titer and increased Th2 profile). Furthermore, the AD model FLG${}^{ft}$/flaky tail mice, which express a reduced amount of dysfunctional truncated protein, performs poorly in discovering molecular commonalities to the human disease [200]. Nevertheless, FLG may be important in specific AD subcohorts (e.g., Eczema herpeticum as depicted in our review) [145].

Investigating the impact of AD on the onset of allergic contact dermatitis revealed an atypical skin response against nickel (Th1-skewed allergenic response) and rubber/fragrance (Th2-skewed allergenic response). In addition to a highly upregulated immunoregulatory response (e.g., *IL37* and *CTLA4*) in AD skin, Correa da Rosa et al. observed an increased Th17-related response and reduced canonical allergen-typical response. In line with their data, an earlier study already stated the role of diverse T effector cell responses in ACD pathophysiology [84]. This finding might have important implications in inducing an appropriate vaccination response in AD patients, especially as AD is highly prevalent in children [201].

## 6. Validating Disease Models

Another intriguing field of transcriptomics is the validation of experimental disease models (e.g., tissue-engineered human psoriatic skin [202]) and the use of molecular profiles of transgenic animal models to investigate the influence of specific genes [203,204]. In this regard, the role of Keratin 1 (*KRT1*) in atopic dermatitis and psoriasis was shown by comparative transcriptomics of the respective murine and human datasets [205]. Through this approach, potential susceptibility genes may be found or suspected genes experimentally validated by assessing the transcriptomic profile of in vitro gene edited cells [135,206]. In a similar way, the role of the cytoskeleton regulator Arp2/3 complex was evaluated by transcriptomics of an *Arpc4* knockout mouse strain to investigate psoriasis-specific features [207]. In the field of psoriasis research, Imiquimod (IMQ)-induced dermatitis is a widely used murine model which mainly acts via TLR7/8 agonism [208] and largely depends on IL-17 signaling [209,210,211]. Swindell et al. investigated its validity in a large study with different mouse strains and gender dependency and compared transcriptomic data with psoriasis and other human inflammatory skin disease profiles. They found a large overlap in transcriptomics of different mouse strains with a variety of diseases (e.g., wounding and infections) reflecting the differential pathophysiology underlying the model and the human disease. Their data suggested C57BL/6 mice as the most accurate IMQ psoriasis model strain [212]. Garzorz-Stark et al., compared human skin treated with IMQ with psoriasis and contact dermatitis: They found that the IMQ-dermatitis transcriptome in human skin rather reflects characteristics of allergic contact dermatitis (e.g., upregulation of IFNα, cytotoxic granules pathway, and apoptosis) instead of psoriasis. On the other hand, they found *NOS2* expression in human IMQ treated skin which is thought to be specific for psoriatic lesions and is usually not found in any type of eczema. The authors therefore concluded the presence of a mainly contact dermatitis-like pathophysiology including pDC activation mimicking partly the current view on the pathogenesis of psoriasis [41].

When additional gene editing or post-transcription interference (i.e., knockdown) methods are applied, complex mechanisms may be illuminated as recently shown for the role of epigenetic hydroxymethylation on *S100A9* expression in the IMQ model [213] or the impact of dermokines (*DMKN*) in skin inflammation and barrier function [214].

The validation of murine models in AD studies is highly needed as no standardized model is currently existing which mimics the highly diverse pathomechanisms (inside-out vs. outside-in theory) [215]. Current AD models include cytokine overexpression (e.g., IL-4 [216] and IL-31), genetic mutations leading to dysfunctional protein expression (e.g., Filaggrin${}^{ft}$/flaky tail mice), NC/Nga model [76,217], PAR2 overexpression and house dust mite (HDM) application [215] and Aspergillus fumigatus-induced AD [200,218]. Formerly used in AD research, the topical application of SDS rather induces characteristics of contact dermatitis [215]. Ewald et al., investigated several murine AD models and compared the transcriptomic profiles with the MADAD [26]. Many of these models only reflect a minor facet of the human disease. Although, in many cases, a model’s inherent characteristic is to reflect a given observed phenomenon only partly, the translation of findings may be highly compromised by separating the interacting features (e.g., dysregulated adaptive immune cells—non-passive keratinocyte immune response), which lead to the complex mechanisms in inflammatory skin diseases.

Not only mouse models are utilized in AD research. As dogs commonly suffer from AD, canine models are considered to mimic the human disease adequately [219,220,221]. Although the role of Th17 related pathways are suggested in AD, investigation of early canine HDM-induced AD lacks Th1 and Th17 activation. A reasonable hypothesis might be the early induction of Th2 pathways with increasing recruitment of Th1 and Th17 in the course of the disease. As itch related *IL31* is highly upregulated as early as six hours after disease induction, a potential mechanism may be the scratch related recruitment of other cellular components (e.g., Th1, Th17, and neutrophils) [220].

In the field of allergic contact dermatitis research, the murine contact hypersensitivity reaction based on a type IV immune response is widely established [222]. Major deficits include the missing disease chronicity and the usage of artificial haptens (e.g., TNCB and DNFB). While these chemicals induce a strong innate response, human contact allergens are commonly weak sensitizer. Recently, Liu et al., introduced a poison ivy-based model (urushiol as the active compound). Thereby, they used a patient-relevant allergen [130].

## 7. Prognostics And Treatment

The determination of gene expression profiles through transcriptome analysis is not only enhancing our understanding of inflammatory skin diseases by uncovering new signaling pathways and related molecular mechanisms, but is also leading to advanced prognostic approaches. In addition to an early evaluation of a treatment response and the detection of a relapse, transcriptomic profiling is likewise promising in understanding so far undefined therapeutic mechanisms on a molecular level. Based on these assumptions, transcriptomics emerges to be pertinent to the establishment of new therapeutic strategies.

Samples from Efalizumab-treated (humanized anti-CD11a) patients were taken prior treatment, at week 12 when the lesions were resolved and in case of a relapse. Interestingly, using an unsupervised clustering algorithm, non-lesional and as effectively treated determined specimens as well as lesional and relapse samples clustered pairwise together stating their respective similarities in molecular profiles [223]. Evaluating treatment response may not only be valuable in systemic treatments but also in radiation-based approaches like narrow band UVB irradiation (NB-UVB) [224] or topical agents like ferulic acid [225], indirubin [43], or rhodomyrtone [226]. For instance, NB-UVB irradiation was shown to exhibit systemic effects by restoring expression of the transcription factor *CEBPB* in PBMCs of treated patients [224].

To determine the treatment response, the respective specimen should be easily accessible (e.g., upper epidermal layer via tape stripping, patient blood/serum). PBMCs of GPP patients showed a prominent “neutrophil activation”-pathway enrichment (e.g., *CXCL1* and *CXCL8*), which markedly normalized upon retinoid treatment [135]. The evaluation of systemically acting mechanisms is a reasonable approach as GPP is accompanied by severe systemic manifestations. These mechanisms might include circulating blood cells or systemically acting cytokines. The direct comparison of PBMCs of psoriasis vulgaris and GPP patients revealed a marked upregulation of genes associated with Type-I IFN production. This upregulation is potentially induced via high amounts of systemically acting IL-36 in GPP [227].

Integrating the before-mentioned analysis of involved master regulatory transcription factors and the pathways activated or inhibited upon treatment, may turn out beneficial in clarifying so far unknown drug effect mechanisms. Through this approach, our understanding of the disease pathophysiology may be indirectly improved [173]. Other treatment studies dissected the transcriptomic data upon IFNγ (fontolizumab) or TNFα (infliximab) inhibition in ex vivo activated PBMCs. Interestingly, in the case of infliximab treatment, they found genes regulated by TNF inhibition that were previously not assigned to the respective signaling pathway like IFNγ or *IL12RB2* [228]. In addition to the classical view on TNF signaling in inducing immediate genes (e.g., *IL1*, *IL6*, and *IL8*), this points to its role in orchestrating multicellular responses by involving the adaptive immune system through for example IFNγ. In the case of adalimumab treatment of psoriasis patients, these changes could be observed gradually in the molecular profile and as early as from day four on [229,230], although TNF inhibitors are known to show a latency in lesion resolution.

When the anti-CD2 antibody alefacept was still listed as an FDA approved drug against psoriasis in the USA, discussions on the value of analyzing the transcriptomic profile of patient groups to classify responders and non-responders took place [14]. Transcriptomic analysis showed that the treatment of psoriasis patients resulted in diverse molecular responses. Additionally, the downregulation of known transcriptomic correlates of active psoriasis (e.g., *IFNγ* or iNOS-encoding *NOS2*) increased the value of the drug at that time [231]. Another case of transcriptomic-guided treatment response evaluation was performed in patients receiving the TNFα-neutralizing biologic etanercept [58]. As depicted in the pathophysiology section of this review, *IL17* as well as *TNFα* are key players in psoriasis. Investigations on the kinetics of the IL-17- and TNFα-induced gene expression revealed their synergistic effects on some genes, which may act as important druggable targets [232,233].

Considering the meta-analysis-derived transcriptome MAD3 as readout, an early effect on keratinocytes was shown by JAK inhibition with tofacitinib. This finding was replicated in an in vitro 3D skin model [234]. Although clinical scoring correlated rather poorly with molecular regression, histologic findings (i.e., KRT16 expression and epidermal thickness) paralleled the observed transcriptomic treatment response in early timepoints [235].

Dupilumab is the first widely approved IL4Rα antagonist, which is effective in suppression of itch-related IL-31 and reduction in dysregulated KRT16 levels in lesional skin [236,237,238]. The transcriptomic response showed improved epidermal differentiation characteristics (e.g., *KRT16* downregulation), improved barrier-related functions (e.g., *FLG*, *LOR*, and *CLDN8* upregulation), and restored lipid metabolism as early as four weeks of treatment. Simultaneously, major changes in Th2 cytokine expression were missing [236,238]. A positive correlation of transcriptomic changes with the clinical presentation as assessed by the EASI score was confirmed [236].

Using the meta-analysis derived atopic dermatitis transcriptome (MADAD) [26] as a reference core AD transcriptome, Pavel et al. presented data which supported the usage of dual JAK/SYK inhibition in AD by showing a dose-dependent improvement of the transcriptomic profile. The rational of this approach refers to the inhibition of T cell polarization by JAK inhibition [239] as well as improvement of the terminal epidermal differentiation by SYK inhibitors [240]. Additionally, *IL31* expression was reduced more efficiently by the dual inhibition approach than that seen in dupilumab treatment. Although recent approaches using pan-JAK inhibition in psoriasis were without a major patient health improvement, this multitarget approach might be worth testing, as Th17 signaling may be targeted more robustly [241]. AD is classically not a Th17 centered disease like psoriasis, but a latent activation of accompanying pathways is seen in disease development [242]. However, the use of ustekinumab in AD showed a rather weak response, although drawing explicit implications is difficult due to bias in the patient treatment status as topical steroid were applied concurrently [243]. Following another treatment regime, the effectiveness of topical application of the phosphodiesterase type 4 inhibitor crisaborol was recently verified in the transcriptomic profile of AD lesions (e.g., upregulation of *IL37*) [244].The investigation of a rather unconventional therapeutic approach by utilizing umbilical cord mesenchymal stem cells showed their immunoregulatory effects after subcutaneous injections in an Aspergillus fulmigatus mouse model [218].

Through evaluation of the skin molecular profile before and after treatment, Suárez-Fariñas et al., came up with the concept of a residual disease genomic profile (RDGP) in treated psoriasis. This phenomenon describes the phenotype transformation of lesional to non-lesional skin, whereas certain genes are persistently differentially expressed. They defined specific genes which orchestrate the observed “molecular scar” (e.g., *LYVE-1, AQP, RAB31, and WNT5A*) upon etanercept treatment [25,32]. As depicted in Figure 2, the presence of a “molecular scar” may represent an alternative baseline in the molecular profile in the pre-inflammatory state of inflammatory skin diseases. Whether currently available treatment options are capable to induce a re-transition to an original healthy state stays elusive.

## 8. Conclusions

As research on transcriptomics in inflammatory skin diseases has been increasing over the last decades, several interesting application fields in clinical and medical science emerged. Molecular profiling enhances the understanding of disease mechanisms, relations between different diseases, and disease subgroups, and comes along with patient-stratified diagnostic and therapeutic approaches. Additionally, transcriptome analyses add another layer of confidence in disease model validation, which potentially improves standardization and increases the level of evidence for drawn conclusions. New possibilities in disease classification such as the subgrouping of psoriatic entities but also conceptualization (e.g., atopic dermatitis endotypes) were introduced and supported by transcriptomic findings. Interestingly, the observed gene-sets often overlap to a certain extend (e.g., pediatric atopic dermatitis—psoriasis). The overlap in gene expression between phenotypically different inflammatory skin diseases points to a common cutaneous response mechanism in the course of inflammation. Skin infiltrating and resident immune cells but also local structural cells (i.e., fibroblasts and keratinocytes) account for these observed changes. In addition to the inflammation-induced common gene expression, disease-specific DEGs lead to the respective molecular and clinical phenotype of different diseases or disease entities (Figure 4). However, only a limited number of inter-disease studies included other diseases than atopic dermatitis and psoriasis.

Future approaches should increasingly focus on the integration of additional dimensions of information as transcriptomics by itself only transmits a limited view on the cellular status. Not all biological functions are reliably reflected by the transcriptome, and may rather be detected in the proteome [110,245,246]. With the integration of genomic information of patients (i.e., GWAS) the functional relevance might be directly evaluated through transcriptomic analyses [247]. In line with this, expression quantitative trait locus (eQTL) analysis is an interesting approach to benefit from available “omic”-datasets. Through integration of genetic variation analysis (e.g., single nucleotide polymorphism detection) and transcriptomic data, eQTL allows to link observed genetic variations and biological function quantitatively. Combined with GWAS, eQTL analysis adds underlying mechanistic information to the description of genetic risk variants [248,249]. This analysis approach is currently evolving but so far infrequently utilized in dermatological research. Other recent studies combined the methylome and transcriptome which allows to correlate epigenetic phenomena to the cellular status [213,248,250].

A major bias in many studies is introduced by the investigation of whole skin biopsies. Transcriptional changes may be under- or over-represented as cutaneous compartments shift in size and architecture during their transition from a healthy to a diseased state. Epidermal hyperplasia may lead to overabundance of the respective epidermis-specific genes whereas dermis-specific genes are underrepresented. This bias leads to false-positive upregulation or false-negative downregulation and holds especially true for low-abundant transcripts which need sensitive technology and careful bioinformatic analysis to overcome dilution effects. An important improvement was made with the application of RNA sequencing in assessing the cellular status through transcriptomics. RNA-Seq, although being more expensive for a long time and requiring a comprehensive skill set in bioinformatic analysis compared to microarray technology, came along with a higher degree of sensitivity and versatility as stated by comparative studies [251]. Comparing datasets from microarray platforms and RNA-Seq approaches is therefore always limited (e.g., lack of Th2 cytokine expression in AD studies [252]). Keeping in mind the potential skin-site dependent transcriptomic baseline, researchers should pay attention to the issue of choosing a proper skin area as a healthy control. Thorough bioinformatic analyses and appropriate choices of controls not just of healthy skin but also of inter-disease controls in combination with “clean” transcriptomic standards gathered from laser capture microscopy studies [36,37] or cell culture of specific cell types enables researcher to address these biases.

More elegantly, single cell RNA sequencing (scRNA-Seq) is going to solve many of the above-mentioned issues. Clusters of so far unknown cell states are going to reveal new subpopulations, and therefore models for conceptualization are highly needed. Datasets are going to be even larger and more difficult to handle but may improve pattern recognition and classifier accuracy. Current methods assessing proteomic features (e.g., FACS, immunostaining, and mass spectrometry) are reproducible in terms of changing tissue preparation or tissue disruption. However, as the transcriptomic level is closer to the core of cellular functionality determined by the actively recruited genetic material, it is also more sensitive and flexible in changes and the cellular status may be already altered after sample preparation for the downstream analysis. Reliable and highly standardized protocols are needed to minimize bias introduced trough experimental methodology and verifying high-throughput results in small specialized experimental settings will be essential to discriminate truth and deception.

## 9. Materials and Methods

**Figure A2:**
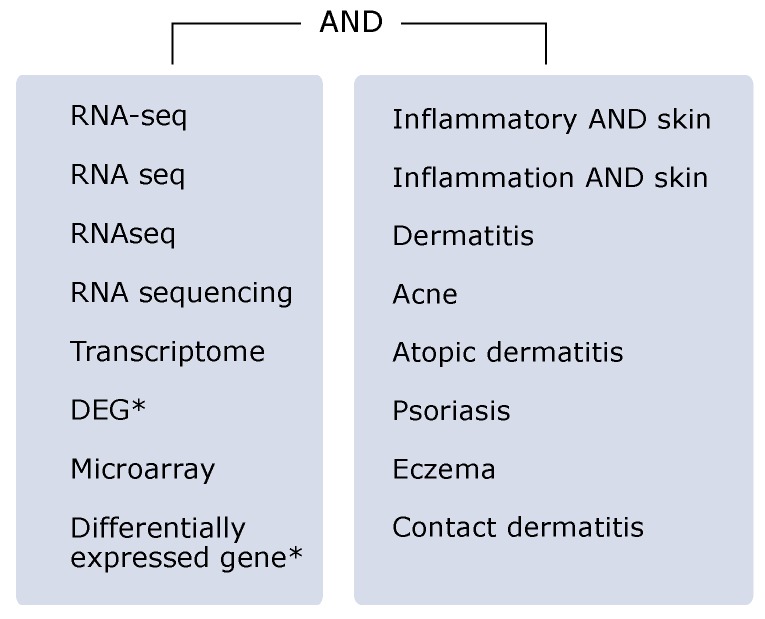
PubMed search term.

**Figure A3:**
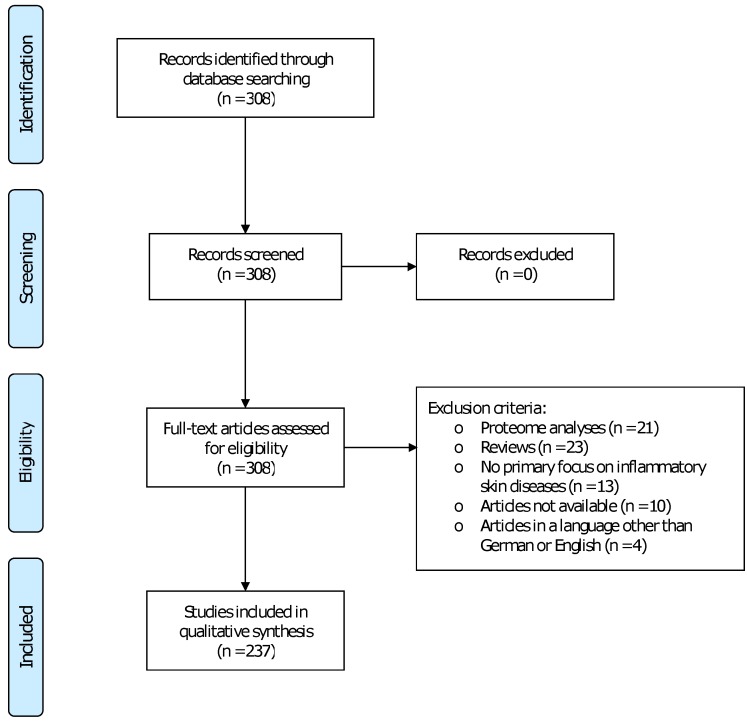
Flowchart of literature search according to PRISMA guidelines.

A systematic PubMed research was conducted using the search term as depicted in Figure A2. Literature selection was performed as stated in the flowchart Figure A3 according to the PRISMA guidelines. Pathway analyses of published transcriptomes MADAD [26] and MAD-5 [25] were carried out using “Enrichr” by the Ma’ayan Lab and Kyoto Encyclopedia of Genes and Genomes 2019 (KEGG2019). Genes depicted in Table A1 represent a selection of the respective pathways.

Landmark papers are listed in Table 1.

## Figures and Tables

**Table 1:** Key papers including important findings. AD = Atopic dermatitis, Pso = Psoriasis, IMQ = IMQ-induced dermatitis, A/ICD = Allergic/irritant contact dermatitis, ADEH = Eczema herpeticum, FA = Food allergy, AB = Asthma bronchiale, EAE = EBI Array Express, NCBI = NCBI Sequence Read Archive, GEO = Gene Expression Omnibus.

Author, Year	Key Message	Disease	Dataset	Study
Fyhrquist et al., 2019	Microbial colonization status allows for AD subgrouping	AD, Pso	NCBI: PRJNA554499, EAE: E-MTAB-8149	[148]
Guttman-Yassky et al., 2019	Dupilumab induces MADAD changes toward a healthy status	AD	Supplementary information	[236]
Kim et al., 2019	STS is a valid sampling method and adequate alternative to whole skin biopsies	AD	NA	[39]
Le et al., 2019	t-SNE analysis does not support an IL-17/IL-22 dual secreting T cell phenotype	Pso	NCBI: SRP165679, SRP026042, SRP057087, SRP035988, SRP050971	[63]
Leung et al., 2019	Pediatric AD patients with or without FA show distinct epidermal molecular profiles	AD, FA	Supplementary information	[110]
Mucha et al., 2019	Multi-omics approach to investigate heritability features of AD	AD	Supplementary information	[49]
Östling et al., 2019	IL-17 high Asthma shows upregulation of psoriasis-like genes and contrasts classical Th2 prone Asthma	AB, Pso	NA	[190]
Pavel et al., 2019	JAK/SYK dual inhibition shows an improved MADAD molecular profile	AD	GEO: GSE133385	[241]
Sanyal et al., 2019	Transcriptomics illuminate different AD endotypes according to patient age and ethnicity	AD	Supplementary information	[119]
Tsoi et al., 2019	AD—Pso cross-disease study shows the proinflammatory state in both diseases. This study states the role of IL-13 in AD.	AD,Pso	GEO: GSE121212	[187]
Ahn et al., 2018	Study defines the core DEG set among different psoriatic entities and highlights distinct profile characteristics	Pso	GEO: GSE117405	[138]
Bin et al., 2018	Study states reduced antiviral response due to ANKRD1 downregulation and increased risk of developing ADEH	ADEH	NA	[146]
Blunder et al., 2018	Cross-disease study of AD and IV questiones the role of *FLG* mutations in AD	AD, IV	GEO: GSE102628	[100]
Brunner et al., 2018	Study illuminates the distict features of pediatric AD, which is skewed toward a Th-17/Th-22 profile	AD	Supplementary information	[118]
Dyjack et al., 2018	Study applies STS to describe different AD endotypes according to the degree of inflammation	AD	Supplementary information	[40]
Meng et al., 2018	IL-31-mediated histamine-independent itch transmission in neuro—immune-axis	AD	NA	[126]
Nattkemper et al., 2018	Study defines the “itchscriptome” as a common gene expression profile of itch in inflammatory skin diseases	AD, Pso	Supplementary information	[128]
Ewald et al., 2017	Study uses human MADAD to validate murine AD models	AD	Supplementary information	[200]
Swindell et al., 2017	Murine IMQ-induced dermatitis shows strain- and gender-dependent molecular profiles. Transcriptomics of IMQ dermatitis mimics rather diverse pathologies and often performs poor in reflecting psoriasic features	Pso	NA	[212]
Bissonnette et al., 2016	Study reasons the distinction of the pustular psoriatic diseases (PPPP and PPP) and states the segregation of PV	Pso	GEO: GSE80047	[141]
Martel et al., 2016	Intrinsic AD profile shows similarities to psoriatic gene expression	AD	GEO: GSE75890	[93]
Varshney et al., 2016	IL-17 signaling in psoriasis is accompanied by downegulated lipid metabolism pathways. Lipid metabolism disturbances reasons for observed pro-atherogenic activity as observed in psoriasis patients	Pso	NA	[85]
Esaki et al., 2015	Identification of skin compartment-specific genes using laser capture microscopy (LCM)	AD	Supplementary information	[36]
Bin et al., 2014	ADEH manifestation due to a disturbed IFNγ response	AD, ADEH	NA	[88]
Dhingra et al., 2014	Molecular profiling reveals an allergen-dependent pathomechanisms in allergic contact dermatitis. Study reasons for disease classification according to the faced allergen	ACD	Supplementary information	[84]
Choy et al., 2012	Description of distinct AD and Pso gene sets and a common lesional profile	AD, Pso	NA	[188]
Mitsui et al., 2012	Definition of skin compartment-specific DEGs utilizing LCM	Pso	GEO: GSE26866	[37]
Tian et al., 2012	Meta-analysis provides psoriasis core transcriptome and description of “molecular scar” after TNF treatment	Pso	NA	[25]
Suárez-Fariñas et al., 2010	Description of a residual disease genomic profile (RDGP) termed “molecular scar” in psoriasis	Pso	GEO: GSE11903	[32]
Sääf et al., 2008	Dysregulated lipid metabolism in atopic dermatitis skin	AD	GEO: GSE12511	[90]

## Abbreviations

The following abbreviations are used in this manuscript:

ACD	Allergic contact dermatitis
AD	Atopic dermatitis
DEG	Differential expressed gene
EDC	Epidermal Differenciation Complex
eQTL	Expression quantitative trait locus
GO	Gene Ontology
GPP	Generalized psoriasis pustulosa
GSEA	Gene Set Enrichment Analysis
IMQ	Imiquimod
IV	Ichthyosis vulgaris
LCM	Laser capture microdissection
MADAD	Meta-analysis derived transcriptome of atopic dermatitis
miRNA	Micro RNA
NET	Neutrophil extracellular trap
PASI	Psoriasis Area and Severity Index
PBMC	Peripheral blood mononuclear cell
PP	Plaque psoriasis
PPAR	Peroxisome proliferator-activated receptor
PPP	Palmo plantar pustulosis
PPPP	Pustular palmo-plantar psoriasis
Pso	Psoriasis
PUVA	Polyunsaturated fatty acid pathway
RDGP	Residual disease genomic profile
RNA-Seq	RNA sequencing
SALT	Skin associated lymphoid tissue
SREBP	Sterol regulatory element-binding protein
STS	Skin tape stripping
TEWL	Trans erpidermal water loss
t-SNE	t-Distributed Stochastic Neighbor Embedding
WGCNA	Weighted Gene Coexpression Network Analysis

## Appendix A

**Table A1:** Pathways and respective GeneSets in Psoriasis transcriptome (MAD-5 [25]) & Atopic dermatitis transcriptome (MADAD) [26]).

MAD-5 [25]	
Pathway	Geneset
PPAR signaling pathway	ADIPOQ, LPL, SORBS1, FADS2, FABP4, ACOX2, FABP5, FABP7, PPARG, ACSBG1, HMGCS2, ANGPTL4, SLC27A2, PPARD
NOD-like receptor signaling pathway	NLRX1, ITPR2, CXCL1, NOD2, CXCL2, IFI16, NAMPT, CASP1, CCL2, IKBKE, STAT1, MAPK14, MAPK13, OAS1/2/3, IL1B, BCL2, IRF7, DEFB4A, MYD88, IRF9
Influenza A	NLRX1, TNFSF10, CASP1, CCL2, IKBKE, HLA-DPA1, RSAD2, PRKCB, DDX58, STAT1, HSPA2, MAPK14, MAPK13, CXCL10, OAS1/2/3, IL1B, IRF7, MYD88, IRF9
Measles	DDX58, STAT1/3, MX1, PIK3R1, HSPA2, IL2RG, CD3D, IFIH1, CCND1, CCNE2, OAS1/2/3, CCNE1, IL1B, BCL2, IRF7, BAK1, BID, IKBKE, MYD88, IRF9, TLR2
Epstein-Barr virus infection	IKBKE, HLA-DPA1, LYN, SYK, STAT1/3, DDX58, TAP2, ISG15, MAPK14, MAPK13, CCNA2, CXCL10, CCNE2, OAS1/2/3, CCNE1, BCL2, IRF7, MYD88, IRF9, TLR2
NF-kappa B signaling pathway	LYN, SYK, DDX58, PRKCB, CXCL2, MALT1, LCK, IL1B, CCL4, LTB, CCL19, CARD14, MYD88, BIRC3
C-type lectin receptor signaling pathway	CCL22, SYK, STAT1, ITPR2, CALML3, NFATC1, PIK3R1, LSP1, MAPK14, MALT1, MAPK13, PYCARD, CYLD, CLEC7A, IL1B, IRF1, BCL3, CASP1, IKBKE, IRF9
Amoebiasis	SERPINB1/3/4/9/13, ARG2, LAMC3, ARG1, PRKCB, LAMB4, LAMC2, CXCL1, PIK3R1, GNA15, PLCB4, IL1B, PLCB1, RAB5A, TLR2
Chemokine signaling pathway	SHC1, CXCR2/4, CXCL1/2/9/10/11/13, CCL2/4/8/18/19/20/22/27 ADCY2, PIK3R1, RAC2, CCR7, YN, PRKCB, STAT1/3, PLCB4, PARD3, PLCB1
IL-17 signaling pathway	MMP1/9, CCL20, CXCL1, CXCL2, MAPK13, CXCL10, LCN2, CCL2, DEFB4A, IKBKE, S100A7/8/9, IL17A
**MADAD [26]**	
Cytokine-cytokine receptor interaction	CCL13, IL20RA, CXCL1, IL2RG, CXCL2, IL27RA, IL18RAP, ACVR1C, CCL8, CCL5, CCL2, CCR7, IL36RN, CCL19, CCL18, IL12RB1, IL13RA2, CCL17, IL12RB2, CCL22, IL4R, TNFRSF12A, IL37, IL36G, EDAR, CXCL10, CXCL11, IL23A, LEP, XCL2, FAS, XCL1, LTB, IL7R, CCL26
Chemokine signaling pathway	LYN, ITK, CCL13, CCL22, STAT1, CXCL1, CXCL2, PIK3CG, PIK3R5, CXCL10, HCK, CXCL11, PLCB4, CCL8, CCL5, XCL2, XCL1, CCL2, CCR7, CCL19, CCL18, JAK3, CCL17, CCL26
PPAR signaling pathway	GK, MMP1, ADIPOQ, AQP7, LPL, FADS2, FABP4, ACADL, ACOX2, FABP7, PLIN4, PPARG, HMGCS2, PLIN1, ANGPTL4
IL-17 signaling pathway	MMP1, MMP3, CXCL1, CXCL2, MMP9, FOSL1, CXCL10, LCN2, CCL2, FOSB, DEFB4A, S100A9, CCL17, S100A8, S100A7
Rheumatoid arthritis	MMP1, IL23A, CCL5, MMP3, CD28, CTLA4, CCL2, CXCL1, ATP6V0A4, LTB, ITGAL, ATP6V1B1
Primary immunodeficiency	PTPRC, LCK, IL2RG, ICOS, IL7R, CD3D, JAK3
JAK-STAT signaling pathway	IL4R, STAT1, FHL1, IL20RA, IL2RG, IL27RA, SOCS3, IL23A, LEP, IL13RA2, IL7R, IL12RB1, STAM2, JAK3, IL12RB2
TNF signaling pathway	SOCS3, CXCL10, IRF1, CCL5, MMP3, CCL2, FAS, CXCL1, NOD2, SELE, CXCL2, MMP9
Cell adhesion molecules (CAMs)	CD274, SELPLG, VTCN1, ITGAL, SELE, CD2, CDH3, PTPRC, SELL, CD6, CLDN8, CD28, CTLA4, ICOS
Inflammatory bowel disease (IBD)	IL18RAP, IL4R, IL23A, STAT1, RORC, NOD2, IL2RG, IL12RB1, IL12RB2

**Table A2:** Studies on MiRNAs in inflammatory skin diseases.

miRNA	Location	Proposed Mechanism	Disease	Study
miRNA-30a-3p	Keratinocytes	Regulation of keratinocyte differentiation via EGFR signaling interaction	Acne inversa	[124]
miRNA-31	Keratinocytes	Interaction with NFκB transcriptional activity	Psoriasis	[253]
	DMSC	Dermal mesenchymal stem cells in psoriasis and impact of miRNAs	Psoriasis	[178]
miRNA-99a	PBMCs	Monitoring treatment response using miRNAs	Psoriasis	[254]
miRNA-143	PBMCs	MiRNA as a biomarker in psoriasis	Psoriasis	[255]
	Keratinocytes	*S. epidermidis* LPA signaling induces miRNA levels which lead to inhibition of *P. acnes*-induced proinflammatory cytokines	Acne	[256]
miRNA-145-5p	Keratinocytes	MiRNA is involved in keratinocyte hyperproliferation and proinflammatory signaling	Psoriasis	[165]
miRNA-146a	PBMCs	Monitoring treatment response using miRNAs	Psoriasis	[254]
miRNA-223	PBMCs	MiRNA as a biomarker in psoriasis	Psoriasis	[255]
miRNA-338-3p	Sebocytes	Suppression of TNF-α-induced lipogenesis in sebocytes	Acne vulgaris	[257]
